# Galactic Cosmic Irradiation Alters Acute and Delayed Species-Typical Behavior in Male and Female Mice

**DOI:** 10.3390/life13051214

**Published:** 2023-05-19

**Authors:** Stephanie Puukila, Olivia Siu, Linda Rubinstein, Candice G. T. Tahimic, Moniece Lowe, Steffy Tabares Ruiz, Ivan Korostenskij, Maya Semel, Janani Iyer, Siddhita D. Mhatre, Yasaman Shirazi-Fard, Joshua S. Alwood, Amber M. Paul, April E. Ronca

**Affiliations:** 1 Oak Ridge Associated Universities, Oak Ridge, TN 37831, USA; 2 NASA, Space Biosciences Division, NASA Ames Research Center, Moffett Field, CA 94035, USA; 3 Space Life Sciences Training Program (SLSTP), NASA Ames Research Center, Moffett Field, CA 94035, USA; 4 Department of Human Factors and Behavioral Neurobiology, Embry-Riddle Aeronautical University, Daytona Beach, FL 32114, USA; 5 Universities Space Research Association, Columbia, MD 21046, USA; 6 The Joseph Sagol Neuroscience Center, Sheba Hospital, Ramat Gan 52621, Israel; 7 Department of Biology, University of North Florida, Jacksonville, FL 32224, USA; 8 Blue Marble Space Institute of Science, Seattle, WA 98154, USA; 9 KBR, Houston, TX 77002, USA; 10 Wake Forest Medical School, Winston-Salem, NC 27101, USA

**Keywords:** GCR radiation exposure, sex differences, rodent behavior, cognition

## Abstract

Exposure to space galactic cosmic radiation is a principal consideration for deep space missions. While the effects of space irradiation on the nervous system are not fully known, studies in animal models have shown that exposure to ionizing radiation can cause neuronal damage and lead to downstream cognitive and behavioral deficits. Cognitive health implications put humans and missions at risk, and with the upcoming Artemis missions in which female crew will play a major role, advance critical analysis of the neurological and performance responses of male and female rodents to space radiation is vital. Here, we tested the hypothesis that simulated Galactic Cosmic Radiation (GCRSim) exposure disrupts species-typical behavior in mice, including burrowing, rearing, grooming, and nest-building that depend upon hippocampal and medial prefrontal cortex circuitry. Behavior comprises a remarkably well-integrated representation of the biology of the whole animal that informs overall neural and physiological status, revealing functional impairment. We conducted a systematic dose-response analysis of mature (6-month-old) male and female mice exposed to either 5, 15, or 50 cGy 5-ion GCRSim (H, Si, He, O, Fe) at the NASA Space Radiation Laboratory (NSRL). Behavioral performance was evaluated at 72 h (acute) and 91-days (delayed) postradiation exposure. Specifically, species-typical behavior patterns comprising burrowing, rearing, and grooming as well as nest building were analyzed. A Neuroscore test battery (spontaneous activity, proprioception, vibrissae touch, limb symmetry, lateral turning, forelimb outstretching, and climbing) was performed at the acute timepoint to investigate early sensorimotor deficits postirradiation exposure. Nest construction, a measure of neurological and organizational function in rodents, was evaluated using a five-stage Likert scale ‘Deacon’ score that ranged from 1 (a low score where the Nestlet is untouched) to 5 (a high score where the Nestlet is completely shredded and shaped into a nest). Differential acute responses were observed in females relative to males with respect to species-typical behavior following 15 cGy exposure while delayed responses were observed in female grooming following 50 cGy exposure. Significant sex differences were observed at both timepoints in nest building. No deficits in sensorimotor behavior were observed via the Neuroscore. This study revealed subtle, sexually dimorphic GCRSim exposure effects on mouse behavior. Our analysis provides a clearer understanding of GCR dose effects on species typical, sensorimotor and organizational behaviors at acute and delayed timeframes postirradiation, thereby setting the stage for the identification of underlying cellular and molecular events.

## 1. Introduction

Exploration beyond the Lower Earth Orbit is a major goal for NASA, international space agencies, and commercial entities. However, the space environment potentially poses significant health risks to astronauts during deep space missions. These space environment stressors include microgravity, ionizing radiation, and social isolation, among others. Immune and central nervous system dysfunction that may arise from exposure to these stressors include altered circadian rhythmicity, anxiety, and degraded cognitive performance [1,2,3,4]. Furthermore, molecular changes induced by space environment stressors can result in detrimental cascade effects [5]. With upcoming missions to the Moon setting the stage for eventual transits to Mars, there is an urgent need to elucidate effects of spaceflight stressors on the central nervous system, in both men and women, which may result in adverse changes in behavior, mood, and performance of critical tasks.

During deep space missions, it is predicted that individual human cells will be traversed by protons every three days, helium nuclei every three weeks, and a heavy ion (Z greater than two) once every three months [6,7]. This radiation exposure may cause adverse cognitive effects on domains of memory, attention, cognitive flexibility, and executive function potentially lasting up to one-year postexposure [8]. A number of studies have shown that exposure to doses ≥25 cGy of single ions (e.g., protons, neutrons ^4^He ^16^O, ^28^Si, ^48^Ti, or ^56^Fe) impairs cognitive functions [9]. Reduced cognitive function from single ion exposure has been accompanied with changes in neuronal morphology and dendritic density [2,10]. However, there remains concerns about whether the cognitive impairments observed from single ion studies are representative of the effects of exposure to the multi-ion, GCR spectrum [9]. Recently, exposure of adult female Wistar rats to 10 cGy simplified GCRsim adversely impacted problem-solving capabilities 14–18 weeks postexposure, where single ion exposure did not. The authors believed this to be due to the higher average LET (or to the higher Z ions−^16^O, ^28^Si or ^56^Fe) of the GCRSim beam compared to the ^4^He ion beam [11]. A further study of the same dose of 10 cGy found that both rats exposed to ^4^He or GCRSim also exhibited poorer performance of fine motor skills about 13 weeks postexposure when compared to sham, and that this was further exasperated with sleep fragmentation [12], However, at 72 h postexposure to 10 cGy of GCRSim or ^4^He, male Wistar rats exhibited poorer performance of fine motor skills when exposed to ^4^He rather than GCRSim [13]. Though, at 12-weeks postirradiation, male Wistar rats exposed to 10 cGy GCRSim showed significant impairment in constrained cognitive flexibility and insightful problem solving [14]. These results suggest that both types of radiation exposure (single ion vs. multi-ion) as well as the timepoint of exposure postanalysis must carefully be considered. Undoubtably, further investigation is critically important to better understand these responses.

For the first time in the past decade, women have comprised ~40% of the traditionally male astronaut classes of NASA. The 2011 National Academy of Sciences Decadal Survey “Recapturing a Future for Space Exploration: Life and Physical Sciences for a New Era” emphasized a need to understand sex differences in spaceflight [15]. In response, the existing literature was reviewed and found that, congruent with Earth-based biomedical findings, sex differences have been reported in space-related studies of cognitive and behavioral performance (summarized by [16]). However, the majority of space radiation studies in rodents have incorporated either one sex or the other. Recent studies [17,18,19,20] revealed profound sexually dimorphic brain and behavioral phenotypes following exposure of male and female mice to single ^4^He ion, 3-ion or 5-ion GCRSim. Male mice exposed to 30 or 50 cGy (^4^He and 3-ion GCRSim, respectively) typically appeared to show more extensive cognitive deficits than females, which was accompanied by increased microglial activation [17,20]. However, females exposed to 50 cGy 5-ion GCRSim exhibited decreased whole-body movement and reduced object recognition. Yet, there was a trend toward an effect of radiation where BDNF levels increased in the cortex of males but not females [19]. Rienceker et al. (2023) observed behavioral deficits and elevated peripheral blood monocytes and Natural Killer cells in group-housed males but not females, after exposure to 50 cGy 5-ion GCRSim [21]. Given the sexually dimorphic nature of the behavioral responses, establishing a GCRSim dose–response curve is key to enabling the selection of a dose that evokes a midrange response in future studies of both sexes to avoid ceiling or floor effects. Additionally, determining long-term physiological responses and adaptation to the space environment is required to ensure the safety and health of astronauts.

Here, we investigated the effects of low-dose radiation using GCRSim on undisturbed species-typical homecage behavior of crew age-matched adult male and female mice. Behavior comprises a remarkably well-integrated representation of the biology of the whole animal that informs overall neural and physiological status. Behavior not only ensures survival and reproductive success; it also provides a means by which the animal can control its environment to promote homeostasis. The study of behavioral changes in mice has been used in countless studies of illness, disease, and disease progression (reviewed by [22]. Homecage analysis has been utilized in models of neurodegenerative diseases, specifically levels of activity, changes in food and water consumption as well as changes in grooming behaviors [22] and has been suggested to be valuable in early diagnosis of neurodegenerative-relevant phenotypes or drug treatment testing [23]. In fact, changes in behavior may be present prior to pathological damage in the brain [24]. Therefore, behavioral analysis can reveal how animals acclimate to the space environment stressors such as irradiation. Species-typical behavior such as burrowing, grooming, rearing, and nest building was analyzed at both early and late postirradiation timepoints. Our data demonstrate sex- and time-specific responses to GCRSim exposure. This study offers novel insight into behavioral responses to spaceflight relevant doses of radiation exposure, indicating potential behavioral/cognitive changes. These potential changes may result in endangered crew health and impact performance during deep space missions.

## 2. Materials and Methods

### 2.1. Animals

Male (*n* = 104) and female (*n* = 104) C57BL/6J mice were purchased from the Jackson Laboratory (Sacramento, CA, USA) and shipped together as a single shipment to Brookhaven Laboratory Animal Facility (BLAF) at Brookhaven National Laboratory (BNL, Upton, NY, USA). Mice were shipped 5 per cage and upon arrival to BNL were housed singly. Animals were acclimated at BNL for 1 week before beginning the study. Males and females were housed in separate rooms for the duration of the study. The animal housing rooms maintained a 12:12 h light:dark cycle, ’~50% humidity and room temperature of 23 ± 2 °C. This study is part of a multiyear project investigating multiple spaceflight stressors, including social isolation. In this study, mice were singly housed to investigate the dose rate effect of GCRSim exposure before evaluating the role of social vs. single housing. Mice were housed singly in standard shoebox cages with standard bedding and a Nestlet (Ancare), replaced weekly. For the duration of the study, mice received food (Teklad 2014, Envigo) and water ad libitum. All animal procedures were approved by the Institutional Animal Care and Use Committees of NASA Ames Research Center (protocol number 516) and BNL (protocol number 516).

Mice were randomly assigned into 4 groups (sham, 5, 15, or 50 cGy; *n* = 13 per group per sex) and two endpoints (intermediate where total *n* = 52 and late where total *n* = 52). The animals in the intermediate group were euthanized at BNL 14 days postradiation exposure. The animals in the late timepoint groups were shipped to the Animal Care Facility at NASA Ames Research Center (Moffett Field, CA, USA) 21 days postexposure. Upon arrival, mice were housed in a reverse 12:12 h light:dark cycle in order to conduct behavioral assays during their active phase and with a room temperature of 23 ± 2 °C. At 126 days following GCRSim exposure, mice were euthanized, and tissues were collected. For all behavioral analyses, the acute timepoint was performed under typical room lighting conditions during the light cycle, while the delayed timepoint was performed under red light during the dark cycle. The acute timepoint behavioral analysis included both endpoint groups, while the delayed behavioral timepoint only included the late endpoint animals. Therefore, for the acute behavioral timepoint, *n* = 26 per group, while for the delayed behavioral timepoint, *n* = 13 per group.

### 2.2. GCRSim Exposure

Mice were exposed to the simplified 5-ion GCRSim protocol [25] at 23–24 weeks of age to simulate a relevant age of astronauts. The 5-ion composition is presented in Table 1. The day before exposure mice were transported to the NASA Space Radiation Laboratory (NSRL) and housed in the animal holding room overnight. Mice underwent a single exposure of 0 (sham), 5 (0.4–0.9 cGy/min), 15 (0.9–1.2 cGy/min), or 50 cGy (1–1.3 cGy/min) using a 60 × 60 cm beam. During each exposure, mice were loaded into 7.3 × 4.0 × 4.0 cm transparent polystyrene restraint boxes with air holes and mounted in the exposure field. Boxes were large enough for mice to turn around. Each box had an absorbent sheet to collect waste during exposure. Animals were placed in the center of the field to assure the best uniformity. The exposure time of the 50 cGy groups ranged from 36 to 43 min. Animals exposed to the lower doses were exposed at the same dose rate and remained in the restraint boxes for the same amount of time as the 50 cGy exposure groups to maintain consistency. Sham animals remained in the restraint boxes for the same amount of time as exposed animals. Animals were returned to their homecages and remained at NSRL until the appropriate radioactivity level was reached, then animals were returned to BLAF.

### 2.3. Composite Garcia Neuroscore

Prior to video acquisition at the acute timepoint (72 h postradiation exposure), a Neuroscore test battery was performed, which assesses functional impairments with a battery of tests following brain injury in rodents [26,27,28]. This composite assessment consists of seven independent subtests which have been previously described [26,29]. Performance and evaluation of each subtest are as follows:

1. Axial sensation (AS): Tactile stimuli was applied on each side of the mouse’s trunk using a cotton swab. Scores indicate the following: 3, mouse is equally startled on both sides; 2, slower response on one side; 1, missing response on one side.
2. Vibrissae proprioception (VP): A cotton swab was moved from the rear of the mouse towards its head and gently touching the vibrissae on each side at a time. Scores indicate: 3, mouse equally turns head on both sides; 2, asymmetric response; 1, missing response on one side.
3. Spontaneous activity (SA): The mouse was observed for 3 min in their homecage environment. Scores indicate the following: 3, mouse approaches at least 3 walls of the cage; 2, mouse approaches less than 3 walls; 1, mouse does not approach any walls; 0, mouse does not move at all.
4. Symmetry of limb movement (LS): The mouse was suspended by the tail to assess movement of the four limbs. Scores indicate: 3, all four limbs were extended symmetrically; 2, asymmetric extension; 1, limbs on one side showed minimal movement; 0, hemiplegia (no limb movement).
5. Lateral turning (LT): The mouse was suspended by the tail and a blunt stick was moved along each side of the body to cause lateral turning towards the stimulus. Scores indicate: 3, animal turns at least 45 degrees on both sides; 2, animal turns equally to both sides but less than 45 degrees; 1, unequal turning; 0, no turning at all.
6. Forelimb outstretching (FO): The mouse was suspended by its tail (<30 s) allowing both forepaws to touch the edge of a table. Scores indicate: 3, forepaws are equally outstretched and the mouse walked symmetrically on forepaws; 2, asymmetric outstretch and impaired forepaw walking; 1, minimal movement of one forepaw; 0, hemiplegia (no limb movement).
7. Climbing (CL): The mouse was placed on a rough surface (22 × 44 cm) at a 45° angle with the table. Scores indicate: 3, mouse climbs to the top of the surface; 2, asymmetric or impaired climbing; 1, mouse fails to climb or showed tendency of circling.

All activity was evaluated manually by the investigator. The final Neuroscore given to each animal is the sum of the seven individual test scores. The minimum score is 3 (worst performance) and the maximum is 21 (best performance).

### 2.4. Homecage Behavioral Analysis

Video recordings were taken at two timepoints, acute (intermediate and late groups) and delayed (just late group) for each animal. The homecage was placed on a table with the camera placed above and pointed directly into the cage below. The feeder and water were removed and a three-minute video was recorded of the mice undisturbed immediately after cage placement. Videos were recorded in the animal holding room with typical overhead lighting. The delayed timepoint recordings were collected in the same way as the acute timepoint but during the dark cycle and with red-bulb overhead lighting.

The total duration of movement, the amount of time spent in the center region of the cage, as well as the frequency and duration of burrowing, rearing, and grooming were scored without knowledge of groups using Ethovision (Noldus) and Behavioral Observation Research Interactive Software [30], respectively. Nestlet development was evaluated from the same three-minute videos using the Deacon score as previously published [31]. In short, each Nestlet is scored on a scale of 1–5, where a score of 1 is depicted by largely untouched cotton square Nestlet and the highest score of 5 is depicted by a fully shredded Nestlet formed into a developed crater shape [31]. Videos were recorded 72 h after a fresh Nestlet was added to the cage.

### 2.5. Statistical Analysis

Statistical analysis was performed using Prism version 9.0.0 and JMP Statistical Software Pro 16.0.0. Data were tested for normality using Shapiro–Wilk. Outliers were identified using ROUT. Depending on the Shapiro–Wilk results, data were analyzed using Two-way ANOVA followed by Tukey post hoc or using Kruskal–Wallis followed by Dunn’s Test. For Deacon score, a chi square test was used to compare the frequency of each Deacon score per dose compared to sham. For time dependent variables, Repeated Measures ANOVA or Mixed Measures analysis was performed. Data are expressed as mean ± SD and a *p* value of <0.05 with a 95% Confidence Interval (CI) was considered as significant.

## 3. Results

### 3.1. Neuroscore

**Figure 1:**
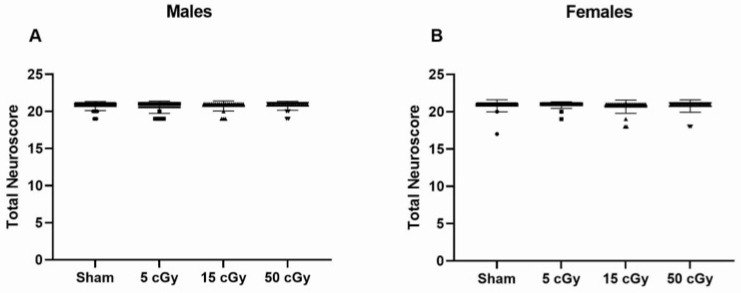
Neuroscore of C57Bl/6J male and female mice exposed to GCRSim. The total of seven tests (spontaneous activity, proprioception, vibrissae touch, limb symmetry, lateral turning, forelimb outstretching, and climbing) was evaluated. Neuroscore for male (**A**) and female (**B**) mice was evaluated 72 h post-GCRSim exposure of either 0 (sham), 5, 15, or 50 cGy. Data are presented as mean ± SD where *n* = 26.

A Neuroscore test battery was performed at the acute timepoint during the light cycle for all animals and the sum of all tests was analyzed. There were no observable deficits in performance of the test battery at any dose and no differences in performance between males (average score 20.7 ± 0.09) and females (average score 20.8 ± 0.08) (Figure 1).

### 3.2. Frequency of Homecage Behaviors

**Figure 2:**
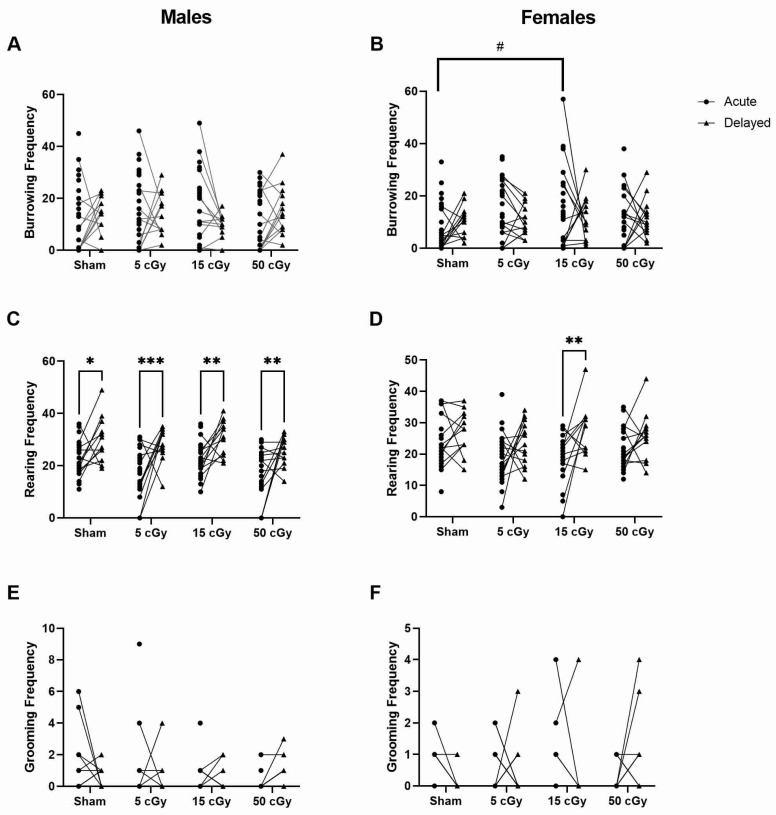
The frequency of typical homecage behavior of C57Bl/6J male and female mice exposed to GCRSim. The total frequency of burrowing (**A**,**B**), rearing (**C**,**D**), and grooming (**E**,**F**) by male and female mice was evaluated in three-minute videos of undisturbed, homecage behavior. Timepoints include 72 h (acute) or 91 days (delayed) post-GCRSim exposure of either 0 (sham), 5, 15, or 50 cGy. Data are presented as individual points where *n* ≥ 13; significance where *p* < 0.05; # denotes dose effects significantly different than sham within that timepoint and * denotes significant difference between acute and delayed timepoints. One symbol denotes *p* < 0.05, two symbols denotes *p* < 0.01, and three symbols denotes *p* < 0.001.

The frequency of typical mouse behaviors such as burrowing, rearing, and grooming were quantified (Figure 2). Many mice did not perform any grooming behavior within the three-minute video timeframe and, therefore, have a frequency value of zero. In the acute exposure groups, during the light cycle, there was no significant difference in any behavior frequencies for the male mice (Figure 2A,C,E). Among the females, we found a significant increase in burrowing frequency in the 15 cGy exposure group compared to the sham (Figure 2B, *p* = 0.0117, 95% CI −15.65 to −1.584). In the delayed exposure groups, during the dark cycle, there were no observable significant differences in any of the behavior frequencies (Figure 2A–F).

When comparing the delayed to the acute timepoint for each dose, the rearing frequency of the male mice increased for all doses (Figure 2C, *p* ≤ 0.0161, 95% CI −14.61 to −1.054 sham, −17.33 to −3.949 5 cGy, −15.28 to −1.720 15 cGy, −15.23 to −2.021 50 cGy), where only 15-cGy-exposed females increased rearing frequency at the delayed timepoint (Figure 2D, *p* = 0.0014, 95% CI −16.35 to −2.974).

For direct comparison of male to female in burrowing, rearing, and grooming frequency, there are no observable differences at any dose or timepoint.

### 3.3. Duration of Homecage Behaviors

**Figure 3:**
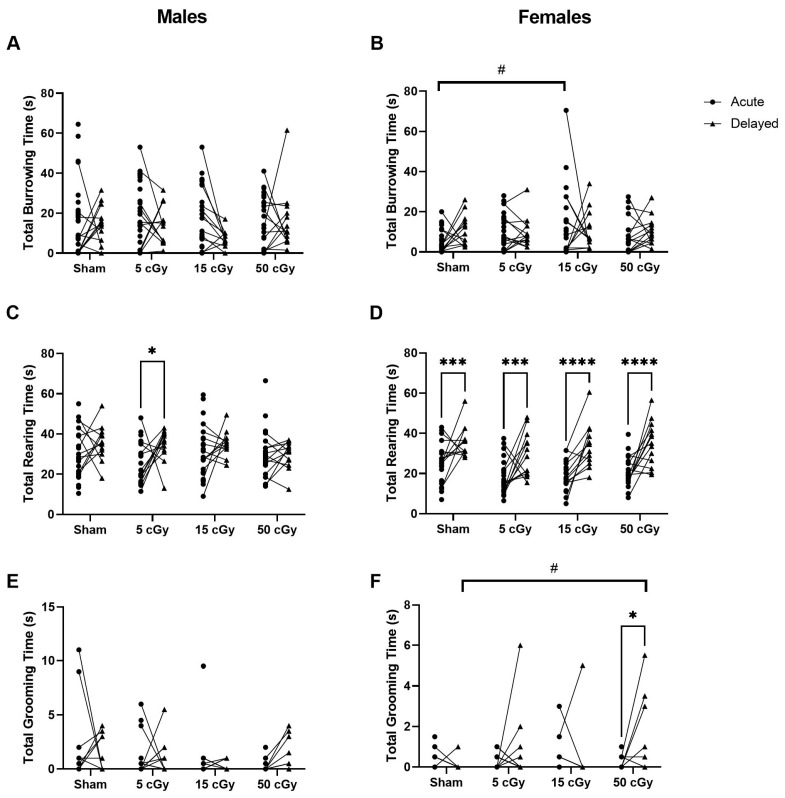
The duration of typical homecage behavior of C57Bl/6J male and female mice exposed to GCRSim. The total duration of burrowing (**A**,**B**), rearing (**C**,**D**), and grooming (**E**,**F**) by male and female mice was evaluated in three-minute videos of undisturbed, homecage behavior. Timepoints include 72 h (acute) or 91 days (delayed) post-GCRSim exposure of either 0 (sham), 5, 15, or 50 cGy. Data are presented as individual points where *n* ≥ 13; significance where *p* < 0.05; # denotes dose effects significantly different than sham within that timepoint and * denotes significant difference between acute and delayed timepoints. One symbol denotes *p* < 0.05, three symbols denotes *p* < 0.001, and four symbols denotes *p* < 0.0001.

The total duration of typical mouse behaviors burrowing, rearing, and grooming was quantified (Figure 3). Many mice did not perform any grooming behavior within the three-minute video timeframe and, therefore, have a duration value of zero. Among the females, we found a significant increase in burrowing duration among the 15 cGy exposure group when compared to the sham at the acute timepoint (Figure 3B, *p* = 0.0127, 95% CI −16.14 to −1.395), similarly as observed with burrowing frequency. There were no other observable differences at the early timepoint. Among the delayed exposure groups, there was no significant difference of male behavior durations among any exposure groups. However, female grooming duration increased after 50 cGy exposure compared to the sham (Figure 3F, *p* = 0.0469, 95% CI −1.908 to −0.009033)

When comparing the delayed to the acute timepoint for each dose, the total rearing duration of male mice exposed to 5 cGy (Figure 3C, *p* = 0.0411, 95% CI −18.96 to −0.2642) and all female mice (Figure 3D, *p* ≤ 0.0004, 95% CI −20.74 to −4.618 sham, −20.04 to −4.541 5 cGy, −23.79 to −7.440 15 cGy, −21.90 to −5.964 50 cGy) was increased. Grooming duration was increased at the delayed timepoint for females exposed to 50 cGy (Figure 3F, *p* = 0.0305, 95% CI −1.676 to −0.05927).

For direct comparison of male to female mice behaviors at the early timepoint, males had a higher burrowing duration than females at all doses except 15 cGy (*p* ≤ 0.0477, 95% CI 2.529 to 21.85 sham, 1.026 to 19.61 5 cGy, 0.06826 to 19.80 50 cGy) and a higher rearing duration than females at all doses except the sham (*p* ≤ 0.0233, 95% CI 0.7464 to 14.83 5 cGy, 5.292 to 20.11 15 cGy, 1.202 to 16.16 50 cGy). There were no observable sex differences in grooming duration. Additionally, at the late timepoint, there were no observable sex differences at any dose for any of the behaviors.

**Figure 4:**
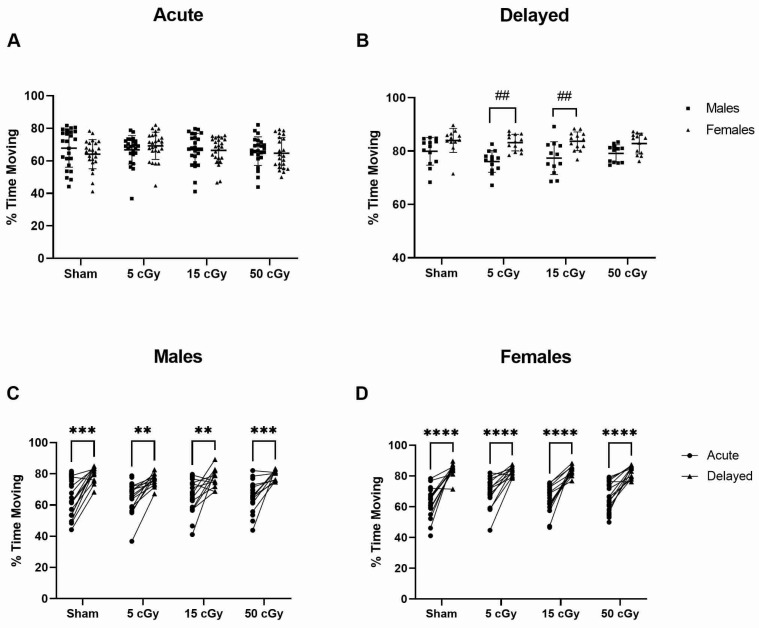
The total duration of movement of C57Bl/6J male and female mice exposed to GCRSim. The total movement duration of male (**A**) and female (**B**) mice was evaluated in 3 min videos of undisturbed, homecage behavior. Timepoints include 72 h (acute; **C**) or 91 days (delayed; **D**) post-GCRSim exposure of either 0 (sham), 5, 15, or 50 cGy. Data are presented as individual points where *n* ≥ 13; significance where *p* < 0.05; # denotes significant difference between sexes at that timepoint and * denotes significant difference between acute and delayed timepoints. Two symbols denotes *p* < 0.01, three symbols denotes *p* < 0.001, and four symbols denotes *p* < 0.0001.

Total duration of movement was quantified (Figure 4). At the delayed timepoint, females spent more time moving than males after 5 and 15 cGy exposure (Figure 4B, *p* = 0.0016, 95% CI −12.31 to −1.835; 0.0095 95% CI −11.65 to −0.9643, respectively). When compared to acute, both males (Figure 4C; *p* ≤ 0.0057, −20.09 to −5.259 sham, −17.00 to −2.259 5 cGy, −18.17 to −3.013 15 cGy, −20.75 to −5.498 50 cGy) and females (Figure 4D *p* < 0.0001, −26.56 to −13.20 sham, −20.37 to −7.535 5 cGy, −23.70 to −10.68 15 cGy, −24.65 to −11.54 50 cGy) of all exposure doses and sham spent more time moving at the delayed timepoint. The velocity, distance traveled, change in heading, and amount of time spent in the center region of the cage was also quantified, but there were no differences between male and females or acute and delayed timepoint.

### 3.4. Deacon Score

**Figure 5:**
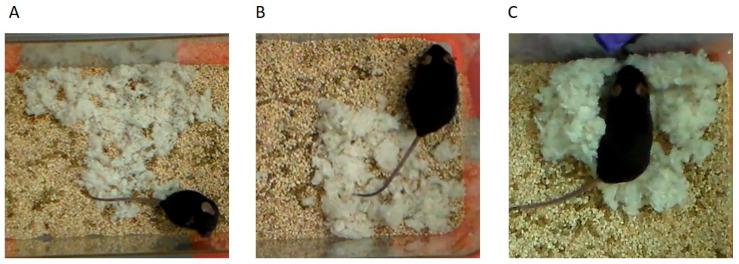
Representative images of Deacon scores of observed Nestlet construction of C57Bl/6J male and female mice exposed to GCRSim. Nestlet construction was evaluated with the Deacon score. In this study, scores were either 3 (**A**), 4 (**B**), or 5 (**C**).

The quality of Nestlet construction was evaluated using the Deacon score (Deacon 2006). In this study, there were no scores of 1 or 2, just 3, 4, and 5 (Figure 5A,B,C).

**Figure 6:**
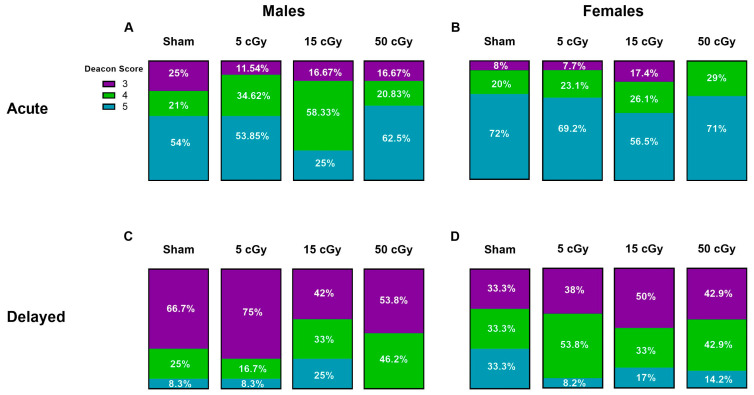
Deacon scores of Nestlet construction of C57Bl/6J male (**A**,**C**) and female (**B**,**D**) mice exposed to GCRSim. The Deacon score of Nestlet construction by male and female mice was evaluated in 3 min videos of undisturbed, homecage behavior. Timepoints include 72 h (acute) (**A**,**B**) or 91 days (delayed) (**C**,**D**) post-GCRSim exposure of either 0 (sham), 5, 15, or 50 cGy. Data are presented as frequency of each score where *n* ≥ 13; significance where *p* < 0.05; # denotes dose effects significantly different than sham within that timepoint and * denotes significant difference between acute and delayed timepoints. One symbol denotes *p* < 0.05.

During the acute observation window, males exposed to the 15 cGy subset had a majority percentage (58.33%) of Nestlet score 4, which was significantly different than the sham, while all other male groups had a majority of score 5 Nestlets (Figure 6A). Interestingly, female mice exposed to 50 cGy had no score 3 Nestlets and only the highest scores of 4 and 5 (Figure 6B). Comparing sex and exposure groups, we found higher overall Deacon scores among the female groups, the majority of scores being a 5, when compared to males. These observations did not remain for the delayed timepoint where there were far fewer Nestlet scores of 5 observed. A majority score of 3 was observed for all doses for males (Figure 6C) and about even scores of 3 and 4 for all females (Figure 6D). In fact, 50 cGy males had no Nestlet score 5 (Figure 6C).

## 4. Discussion

The exposure to spaceflight may result in cognitive deficits that impact astronauts’ behavior and mood. This study, using a mouse model, focused on typical, undisturbed homecage behavior at 72 h (acute) and 91-day (delayed) timepoints following a single dose exposure of sham, 5, 15, or 50 cGy GCRSim. The use of undisturbed homecage video to evaluate behavior has increased in popularity due to ease of performance and low cost. An important consideration is that animals, particularly mice, have a great propensity to compensate in behavior when deficits occur. As the homecage behavior that was assessed are behaviors that mice are able to perform all the time, they may have a greater ability to compensate over time. This would make it more difficult to find differences between groups in the homecage after exposure to space radiation, especially at a delayed timepoint. However, homecage analysis of species-typical behavior is still a valuable tool to investigate GCRSim-induced changes. It is beneficial over out-of-cage testing as it removes any variability induced by the stress of being removed and outside of a familiar homecage, as well as potentially identifying any behavioral patterns that may have been missed during out-of-cage testing [32,33].

Burrowing, rearing, and grooming are naturalistic mouse behaviors, though increases in these behaviors are associated with anxiety and compulsion [34,35,36]. Additionally, changes in these behaviors appear to be sensitive to acute stress and are sex dependent [36]. Here, we observed significant burrowing frequency and duration differences among the female exposure groups during the acute observation period (72 h after exposure) after 15 cGy exposure. The only delayed response differences observed was female grooming duration increased after 50 cGy exposure. A reason for this observation might be that due to such an immediate evaluation window following radiation exposure at the acute timepoint, there may be an adjustment period that results in changing behavior durations due to a recent change in environment (from the restraint boxes during irradiation to the homecage) and that female mice are more sensitive to these changes. Additionally, females exposed to 5 and 15 cGy spent more time moving than males, but only at the delayed timepoint. Previously, in open field, Raber et al. observed that females had higher activity levels than males, but for all doses and sham exposures [18], where Lui et al. found male mice were more susceptible to radiation-induced changes in activity levels than female mice after 10 or 50 cGy exposure of ^56^Fe [37]. Rienecker et al. did not observe any dose or sex differences in the time spent in the center of the open field arena [21]. Here, both males and females spent more time moving at the delayed timepoint than acute, regardless of dose. Age may play a factor in the differences observed between the acute and delayed timepoints. Age-related changes have been observed in locomotion and spatial orientation in rodents [38]. Another important consideration is that the acute timepoint videos were recorded in the light cycle while the delayed timepoint videos were recorded during the dark cycle, and this is likely the reason for increased movement at the delayed timepoint. Both male and female homecage activity has been shown to increase in the dark cycle compared to light, regardless of radiation dose [19]. This may also be the reason rearing differences were observed in both males and females at all exposure doses including sham. The effects of the light:dark cycle on mouse behaviors have been investigated [39,40,41]; however, why only rearing was affected in our study and not burrowing or grooming is unknown and requires further investigation. These results also differed from those observed previously, where male mice exposed to 50 cGy have been shown to have higher activity levels than sham mice during the dark cycle [18]. However, mice in this study were exposed via 3-ion GCRSim rather than the 5-ion. Exposures to the 5-ion GCRSim did not affect homecage activity [19]. In the open field, mice irradiated with 25 cGy 3-ion exposure showed increased activity while mice irradiated with 50 cGy 5-ion exposure showed decreased activity [18,19]. These results indicate a need for further investigation in multi-ion exposures to identify behavioral changes that arise following exposure to the full GCR spectrum experienced in spaceflight. In our study, as each timepoint was evaluated under differing light cycle conditions, it is difficult to interpret the changes in behavior from the acute to the delayed timepoint. However, very few radiation-induced effects were observed, and this response is likely not dependent on the lighting conditions at the time of behavioral analysis.

Nesting is also a naturalistic, highly motivated mouse behavior. Stress exposure impairs nesting in male and female mice [42,43,44,45,46,47,48]. The Deacon score is also a low cost, noninvasive method to evaluate potentially previously unknown behavioral phenotypes. Normal mice are expected to score 3 and above [31], while mice with hippocampal lesions score on average 2 [31,49]. In our study, females were observed to have more frequent high Deacon scores (score of 4 or 5) both during the 72 h and 91-day observation periods. Sex differences in nest building have been observed previously [48,50,51], with females typically performing better than males. This is to be expected, as females are evolutionarily more highly motivated to nest build for the birth and care of pups. Interestingly, females exposed to 50 cGy was the only group to have no Nestlet scores below 4. It is not immediately clear why; though, perhaps 50 cGy exposure induced a mild stress response, as mild stress has been shown to increase nesting in females and not males [52]; however, further investigation is needed.

Analysis during the delayed observation window showed a decline in Nestlet builds (score 4, 5) across all sexes and exposure groups with an increase in frequency of Deacon scores of 3. Most notably, males in the 50 cGy exposure group had no score 5 Nestlets observed. There are a number of potential reasons for this: The delayed animals were housed in a different facility, though the same level of husbandry and the same Nestlet style were provided. The delayed mice experienced more handling than the acute mice as additional testing was performed (not presented here). However, all mice in the delayed timepoint experienced the same amount of handling, so this is not expected to affect the dose response. With acute to delayed timepoint comparisons, as very few radiation-induced effects were observed, we do not expect additional handling to have a strong effect on the delayed response to GCRSim exposure. Additionally, age-induced decline in nest building has been observed in both male and female mice [51], where mice 18 months older built lower scoring Nestlets. Here, mice at the delayed timepoint were only three months older than at the acute timepoint, so it is not abundantly clear if our observed decline in Nestlet scores was age-induced. It is also important to remember that normal mice are expected to score at least 3 [31], and though different from acute, our observations at the delayed timepoint are still considered normal behavior.

Sensorimotor deficits have been observed after exposure of ^56^Fe at similar doses used in this study at 72 h postexposure in male Sprague-Dawley rats [53]. Here, to assess motor and behavioral deficits, a noninvasive test battery (Neuroscore) was performed to assay sensorimotor integrity by evaluating spontaneous activity, proprioception, vibrissae touch, limb symmetry, lateral turning, forelimb outstretching, and climbing at the acute timepoint. We observed no deficits in test score, suggesting no sensorimotor impairments from these doses, though homecage behavior and nest building analysis revealed apparent sexually dimorphic dose responses. This lack of Neuroscore effects emphasizes the sensitivity of homecage analysis to capture the complexity of mouse behavior in response to spaceflight relevant doses of radiation exposure. It is also possible that each of the separate individual tasks within the Neuroscore test were not sensitive enough to pick up the integrated deficits that are unveiled with highly organized, complex behavior, such as nest building and previously reported string-pulling behavior. As previously seen, different behavioral tests can result in different outcomes, even with the same exposure dose [11,13,14]. The homecage is thought to be important for identifying the behavioral patterns that may have been otherwise missed by out-of-cage analysis [33]. Therefore, homecage analysis may be required to fully characterize the postirradiation exposure mouse behavioral phenotype.

A major limitation of this study is that only three-minute videos were utilized. Continuous video acquisition and analysis allows for more accurate monitoring, particularly during the dark cycle when mice are most active [54,55,56], as well as offering insight into previously unobserved activity phenotypes and allow the researcher to fully characterize the behavior [33,39]. Additionally, circadian rhythm, an important component of animal behavior, was not analyzed. Recently, a study by Pernold et al. (2021) found highly significant circannual oscillation in the spontaneous activity of male and female C57BL/6 mice housed under constant conditions. These oscillations in rhythmicity lasted for months and these rhythmic variations likely have a significant effect on undisturbed, homecage behavior [57]. Our future studies aim to investigate the combined effects of numerous spaceflight-like environmental stressors. This will include utilizing hindlimb unloading as a model of microgravity as well as social and single housing and 5-ion GCRSim exposure.

## 5. Conclusions

Overall, in our study, there were some but very few effects of GCRSim exposure on behavior, particularly at 15 and 50 cGy. Though it did not appear that a single dose exposure induced strong negative effects on homecage behavior in mice. Additionally, it does not appear that GCRSim exposure has major negative impacts on Nestlet construction. Further studies are needed to elucidate the effects of galactic cosmic radiation on cognition and behavior that may impact astronaut performance.

## Figures and Tables

**Table 1:** Definition of the simplified GCR simulation in order of delivery [25].

Ion	Energy (MeV/n)	Fraction
H	1000	35%
Si	600	1%
He	250	18%
O	350	6%
Fe	600	1%
H	250	39%

## Data Availability

Data will be made available on NASA open-source archives.

## References

[1] Euston D.R., Gruber A.J., McNaughton B.L. (2012). The Role of Medial Prefrontal Cortex in Memory and Decision Making. Neuron.

[2] Parihar V.K., Allen B., Tran K.K., Macaraeg T.G., Chu E.M., Kwok S.F., Chmielewski N.N., Craver B.M., Baulch J.E., Acharya M.M. (2015). What Happens to Your Brain on the Way to Mars. Sci. Adv..

[3] Parihar V.K., Maroso M., Syage A., Allen B.D., Angulo M.C., Soltesz I., Limoli C.L. (2018). Persistent Nature of Alterations in Cognition and Neuronal Circuit Excitability after Exposure to Simulated Cosmic Radiation in Mice. Exp. Neurol..

[4] Raber J., Torres E.R.S., Akinyeke T., Lee J., Boutros S.J.W., Turker M.S., Kronenberg A. (2018). Detrimental Effects of Helium Ion Irradiation on Cognitive Performance and Cortical Levels of MAP-2 in B6D2F1 Mice. Int. J. Mol. Sci..

[5] Mhatre S.D., Iyer J., Puukila S., Paul A.M., Tahimic C.G.T., Rubinstein L., Lowe M., Alwood J.S., Sowa M.B., Bhattacharya S. (2022). Neuro-Consequences of the Spaceflight Environment. Neurosci. Biobehav. Rev..

[6] Krukowski K., Grue K., Becker M., Elizarraras E., Frias E.S., Halvorsen A., Koenig-Zanoff M., Frattini V., Nimmagadda H., Feng X. (2021). The Impact of Deep Space Radiation on Cognitive Performance: From Biological Sex to Biomarkers to Countermeasures. Sci. Adv..

[7] Nelson G.A. (2016). Space Radiation and Human Exposures, A Primer. Radiat. Res..

[8] Jandial R., Hoshide R., Waters J.D., Limoli C.L. (2018). Space-Brain: The Negative Effects of Space Exposure on the Central Nervous System. Surg. Neurol. Int..

[9] Huff J.L., Poignant F., Rahmanian S., Khan N., Blakely E.A., Britten R.A., Chang P., Fornace A.J., Hada M., Kronenberg A. (2023). Galactic Cosmic Ray Simulation at the NASA Space Radiation Laboratory—Progress, Challenges and Recommendations on Mixed-Field Effects. Life Sci. Space Res..

[10] Parihar V.K., Allen B.D., Caressi C., Kwok S., Chu E., Tran K.K., Chmielewski N.N., Giedzinski E., Acharya M.M., Britten R.A. (2016). Cosmic Radiation Exposure and Persistent Cognitive Dysfunction. Sci. Rep..

[11] Britten R.A., Fesshaye A., Ihle P., Wheeler A., Baulch J.E., Limoli C.L., Stark C.E. (2022). Dissecting Differential Complex Behavioral Responses to Simulated Space Radiation Exposures. Radiat. Res..

[12] Blackwell A.A., Tracz J.A., Fesshaye A.S., Tidmore A., Osterlund Oltmanns J.R., Schaeffer E.A., Lake R.I., Wallace D.G., Britten R.A. (2023). Fine Motor Deficits Exhibited in Rat String-Pulling Behavior Following Exposure to Sleep Fragmentation and Deep Space Radiation. Exp. Brain Res..

[13] Blackwell A.A., Fesshaye A., Tidmore A., Lake R.I., Wallace D.G., Britten R.A. (2022). Rapid Loss of Fine Motor Skills after Low Dose Space Radiation Exposure. Behav. Brain Res..

[14] Britten R.A., Fesshaye A., Tidmore A., Blackwell A.A. (2022). Similar Loss of Executive Function Performance after Exposure to Low (10 CGy) Doses of Single (4He) Ions and the Multi-Ion GCRSim Beam. Radiat. Res..

[15] Council N.R. (2011). Recapturing a Future for Space Exploration: Life and Physical Sciences Research for a New Era. Recapturing a Future for Space Exploration: Life and Physical Sciences Research for a New Era.

[16] Mark S., Scott G.B.I., Donoviel D.B., Leveton L.B., Mahoney E., Charles J.B., Siegel B. (2014). The Impact of Sex and Gender on Adaptation to Space: Executive Summary. J. Womens Health.

[17] Parihar V.K., Angulo M.C., Allen B.D., Syage A., Usmani M.T., Passerat de la Chapelle E., Amin A.N., Flores L., Lin X., Giedzinski E. (2020). Sex-Specific Cognitive Deficits Following Space Radiation Exposure. Front. Behav. Neurosci..

[18] Raber J., Yamazaki J., Torres E.R.S., Kirchoff N., Stagaman K., Sharpton T., Turker M.S., Kronenberg A. (2019). Combined Effects of Three High-Energy Charged Particle Beams Important for Space Flight on Brain, Behavioral and Cognitive Endpoints in B6D2F1 Female and Male Mice. Front. Physiol..

[19] Raber J., Fuentes Anaya A., Torres E.R.S., Lee J., Boutros S., Grygoryev D., Hammer A., Kasschau K.D., Sharpton T.J., Turker M.S. (2020). Effects of Six Sequential Charged Particle Beams on Behavioral and Cognitive Performance in B6D2F1 Female and Male Mice. Front. Physiol..

[20] Krukowski K., Grue K., Frias E.S., Pietrykowski J., Jones T., Nelson G., Rosi S. (2018). Female Mice Are Protected from Space Radiation-Induced Maladaptive Responses. Brain Behav. Immun..

[21] Rienecker K.D.A., Grue K., Paladini M.S., Frias E.S., Frattini V., Borlongan M.C., Chou A., Torres-Espin A., Krukowski K., Ferguson A.R. (2023). Combined Space Stressors Induce Independent Behavioral Deficits Predicted by Early Peripheral Blood Monocytes. Sci. Rep..

[22] Richardson C.A. (2015). The Power of Automated Behavioural Homecage Technologies in Characterizing Disease Progression in Laboratory Mice: A Review. Appl. Anim. Behav. Sci..

[23] Si Y., Guo C., Xiao F., Mei B., Meng B. (2022). Noncognitive Species-Typical and Home-Cage Behavioral Alterations in Conditional Presenilin 1/Presenilin 2 Double Knockout Mice. Behav. Brain Res..

[24] Codita A., Gumucio A., Lannfelt L., Gellerfors P., Winblad B., Mohammed A.H., Nilsson L.N.G. (2010). Impaired Behavior of Female Tg-ArcSwe APP Mice in the IntelliCage: A Longitudinal Study. Behav. Brain Res..

[25] BNL|NSRL User Guide. https://www.bnl.gov/nsrl/userguide/simgcrsim.php.

[26] Garcia J.H., Wagner S., Liu K.F., Hu X.J. (1995). Neurological Deficit and Extent of Neuronal Necrosis Attributable to Middle Cerebral Artery Occlusion in Rats. Statistical Validation. Stroke.

[27] Hausser N., Johnson K., Parsley M.A., Guptarak J., Spratt H., Sell S.L. (2018). Detecting Behavioral Deficits in Rats after Traumatic Brain Injury. J. Vis. Exp..

[28] McBride D.W., Wang Y., Adam L., Oudin G., Louis J.S., Tang J., Zhang J.H. (2016). Correlation Between Subacute Sensorimotor Deficits and Brain Edema in Rats after Surgical Brain Injury. Acta Neurochir. Suppl..

[29] Krafft P.R., McBride D.W., Lekic T., Rolland W.B., Mansell C.E., Ma Q., Tang J., Zhang J.H. (2014). Correlation between Subacute Sensorimotor Deficits and Brain Edema in Two Mouse Models of Intracerebral Hemorrhage. Behav. Brain Res..

[30] Friard O., Gamba M. (2016). BORIS: A Free, Versatile Open-Source Event-Logging Software for Video/Audio Coding and Live Observations. Methods Ecol. Evol..

[31] Deacon R.M.J. (2006). Assessing Nest Building in Mice. Nat. Protoc..

[32] Balzani E., Falappa M., Balci F., Tucci V. (2018). An Approach to Monitoring Home-Cage Behavior in Mice That Facilitates Data Sharing. Nat. Protoc..

[33] Voikar V., Gaburro S. (2020). Three Pillars of Automated Home-Cage Phenotyping of Mice: Novel Findings, Refinement, and Reproducibility Based on Literature and Experience. Front. Behav. Neurosci..

[34] Pond H.L., Heller A.T., Gural B.M., McKissick O.P., Wilkinson M.K., Manzini M.C. (2021). Digging Behavior Discrimination Test to Probe Burrowing and Exploratory Digging in Male and Female Mice. J. Neurosci. Res..

[35] Kalueff A.V., Stewart A.M., Song C., Berridge K.C., Graybiel A.M., Fentress J.C. (2016). Neurobiology of Rodent Self-Grooming and Its Value for Translational Neuroscience. Nat. Rev. Neurosci..

[36] Sturman O., Germain P.L., Bohacek J. (2018). Exploratory Rearing: A Context- and Stress-Sensitive Behavior Recorded in the Open-Field Test. Stress.

[37] Liu B., Hinshaw R.G., Le K.X., Park M.A., Wang S., Belanger A.P., Dubey S., Frost J.L., Shi Q., Holton P. (2019). Space-like 56Fe Irradiation Manifests Mild, Early Sex-Specific Behavioral and Neuropathological Changes in Wildtype and Alzheimer’s-like Transgenic Mice. Sci. Rep..

[38] Osterlund Oltmanns J.R., Schaeffer E.A., Blackwell A.A., Lake R.I., Einhaus R.M., Kartje G.L., Wallace D.G. (2022). Age-Related Changes in the Organization of Spontaneously Occurring Behaviors. Behav. Process..

[39] Bains R.S., Wells S., Sillito R.R., Armstrong J.D., Cater H.L., Banks G., Nolan P.M. (2018). Assessing Mouse Behaviour throughout the Light/Dark Cycle Using Automated in-Cage Analysis Tools. J. Neurosci. Methods.

[40] Peirson S.N., Brown L.A., Pothecary C.A., Benson L.A., Fisk A.S. (2018). Light and the Laboratory Mouse. J. Neurosci. Methods.

[41] Steel L.C.E., Tir S., Tam S.K.E., Bussell J.N., Spitschan M., Foster R.G., Peirson S.N. (2022). Effects of Cage Position and Light Transmission on Home Cage Activity and Circadian Entrainment in Mice. Front. Neurosci..

[42] Dournes C., Beeské S., Belzung C., Griebel G. (2013). Deep Brain Stimulation in Treatment-Resistant Depression in Mice: Comparison with the CRF1 Antagonist, SSR125543. Prog. Neuropsychopharmacol. Biol. Psychiatry.

[43] Otabi H., Goto T., Okayama T., Kohari D., Toyoda A. (2016). Subchronic and Mild Social Defeat Stress Alter Mouse Nest Building Behavior. Behav. Process..

[44] Otabi H., Goto T., Okayama T., Kohari D., Toyoda A. (2017). The Acute Social Defeat Stress and Nest-Building Test Paradigm: A Potential New Method to Screen Drugs for Depressive-like Symptoms. Behav. Process..

[45] Woo H., Hong C.J., Jung S., Choe S., Yu S.W. (2018). Chronic Restraint Stress Induces Hippocampal Memory Deficits by Impairing Insulin Signaling. Mol. Brain.

[46] Gjendal K., Ottesen J.L., Olsson I.A.S., Sørensen D.B. (2019). Burrowing and Nest Building Activity in Mice after Exposure to Grid Floor, Isoflurane or Ip Injections. Physiol. Behav..

[47] Newman E.L., Covington H.E., Suh J., Bicakci M.B., Ressler K.J., DeBold J.F., Miczek K.A. (2019). Fighting Females: Neural and Behavioral Consequences of Social Defeat Stress in Female Mice. Biol. Psychiatry.

[48] Jacobson M.L., Wulf H.A., Tsuda M.C., Browne C.A., Lucki I. (2020). Sex Differences in the Modulation of Mouse Nest Building Behavior by Kappa Opioid Receptor Signaling. Neuropharmacology.

[49] Cunningham C., Deacon R., Wells H., Boche D., Waters S., Picanco Diniz C., Scott H., Rawlins J.N.P., Perry V.H. (2003). Synaptic Changes Characterize Early Behavioural Signs in the ME7 Model of Murine Prion Disease. Eur. J. Neurosci..

[50] Reiber M., Koska I., Pace C., Schönhoff K., von Schumann L., Palme R., Potschka H. (2022). Development of Behavioral Patterns in Young C57BL/6J Mice: A Home Cage-Based Study. Sci. Rep..

[51] Xiong X.D., Xiong W.D., Xiong S.S., Chen G.H. (2018). Age- and Gender-Based Differences in Nest-Building Behavior and Learning and Memory Performance Measured Using a Radial Six-Armed Water Maze in C57BL/6 Mice. Behav. Neurol..

[52] Lippi S.L.P. (2021). Chronic Mild Unpredictable Stress and High-Fat Diet Given during Adolescence Impact Both Cognitive and Noncognitive Behaviors in Young Adult Mice. Brain Sci..

[53] Joseph J.A., Hunt W.A., Rabin B.M., Dalton T.K. (1992). Possible Accelerated Striatal Aging Induced by 56Fe Heavy-Particle Irradiation: Implications for Manned Space Flights. Radiat. Res..

[54] Howerton C.L., Garner J.P., Mench J.A. (2012). A System Utilizing Radio Frequency Identification (RFID) Technology to Monitor Individual Rodent Behavior in Complex Social Settings. J. Neurosci. Methods.

[55] Steele A.D., Jackson W.S., King O.D., Lindquist S. (2007). The Power of Automated High-Resolution Behavior Analysis Revealed by Its Application to Mouse Models of Huntington’s and Prion Diseases. Proc. Natl. Acad. Sci. USA.

[56] Singh S., Bermudez-Contreras E., Nazari M., Sutherland R.J., Mohajerani M.H. (2019). Low-Cost Solution for Rodent Home-Cage Behaviour Monitoring. PLoS ONE.

[57] Pernold K., Rullman E., Ulfhake B. (2021). Major Oscillations in Spontaneous Home-Cage Activity in C57BL/6 Mice Housed under Constant Conditions. Sci. Rep..

